# Reduced Modulation of Theta and Beta Oscillations Mediates Empathy Impairment in Parkinson's Disease

**DOI:** 10.1002/brb3.70294

**Published:** 2025-02-16

**Authors:** Jinying Han, Liuzhenxiong Yu, Mengqi Wang, Xin Chen, Ziye Zhao, Pingping Liu, Lili Hu, Lingling Lv, Fengbo Xing, Ruihua Cao, Rong Ye, Kai Wang, Panpan Hu

**Affiliations:** ^1^ Department of Neurology The First Affiliated Hospital of Anhui Medical University Hefei China; ^2^ School of Mental Health and Psychological Sciences Anhui Medical University Hefei China; ^3^ Anhui Province Key Laboratory of Cognition and Neuropsychiatric Disorders Hefei China; ^4^ Collaborative Innovation Centre of Neuropsychiatric Disorder and Mental Health Hefei China; ^5^ Institute of Artificial Intelligence, Hefei Comprehensive National Science Center Hefei China; ^6^ Anhui Institute of Translational Medicine Hefei China

**Keywords:** empathy, event‐related desynchronization, event‐related synchronization, Parkinson's disease, time–frequency analysis

## Abstract

**Background:**

Empathy is an inaccessible part of advanced social cognitive functions in humans. Impairment of empathy greatly affects the quality of life of patients with Parkinson's disease (PD) but the underlying neurophysiologic mechanisms have not been established.

**Objectives:**

The dynamic process of brain oscillations in PD pain empathy was explored and the mechanism of empathy damage was studied.

**Methods:**

A total of 27 patients with PD and 13 healthy controls were recruited to undergo a pain judgment task, and the event‐related potentials were recorded. This study compared the changes in theta and beta oscillations among two groups after the presentation of painful and neutral stimuli.

**Results:**

Time–frequency analysis results revealed that patients with PD exhibited event‐related theta oscillation synchronization and beta oscillation desynchronization during pain empathy. Compared to healthy controls, patients with PD exhibited a reduced magnitude of beta oscillation desynchronization in response to painful stimuli and attenuated synchronization of theta oscillations induced by neutral stimuli. There are abnormal beta power differences between painful and neutral stimuli, while no differences were found in theta power in PD. Moreover, a positive correlation existed between the degree of beta oscillation desynchronization associated with painful stimuli and the accuracy of pain judgments.

**Conclusion:**

Pain empathy deficits in PD were associated with reduced dynamic modulation of brain theta and beta oscillations.

## Graphical abstract

In our study on pain empathy in PD, we observed that the event‐related synchronization/desynchronization (ERS/ERD) of theta and beta oscillations in PD patients was significantly reduced compared to HCs. Moreover, the event‐related power differences between painful and neutral stimuli exhibited distinct patterns.

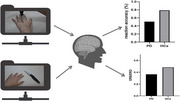

## 1 Introduction

Parkinson's disease (PD) is a neurodegenerative disorder involving motor and nonmotor symptoms (Tolosa et al. 2021). It is well‐known that motor symptoms slow mobility or even render patients with PD paralyzed in bed, making self‐care difficult. Although nonmotor symptoms do not affect the lives of patients with PD to the same extent as motor symptoms, nonmotor symptoms have a high incidence (Zhu et al. 2024). Among these, impairments in empathy have been frequently reported, particularly in the context of emotional and social processing (Alonso‐Recio et al. 2021; Argaud et al. 2018). Such deficits may manifest as diminished emotional resonance with others' distress or as difficulties in perspective‐taking, potentially leading to reduced prosocial behavior and poorer quality of life for patients and their caregivers (Coundouris, Adams, and Henry 2020). Despite their clinical relevance, the neural mechanisms underlying empathy deficits in PD, particularly in the domain of pain empathy, remain poorly understood.

Empathy, a fundamental attribute of social cognition, refers to inferring or feeling the emotional experiences of others (Völlm et al. 2006). Empathy involves the process of sharing, understanding, and responding to the emotions of others (Gallagher, Raffone, and Aglioti 2024), and it is composed of three independent but related components. These components mainly include affective, cognitive, and motivational components (Weisz and Cikara 2021). Affective empathy, also known as an emotional component or experience sharing, involves indirectly experiencing the emotional states of others. The cognitive component, or perspective‐taking, includes understanding others' thoughts or experiences. Finally, the motivational component, which encompasses compassion or empathic concern, involves a desire to promote the well‐being of others (Weisz and Cikara 2021; Bartochowski et al. 2018). According to the empathy model proposed by Goubert et al. (2005), the bottom‐up emotional component occurs in the early stages, after which top‐down cognitive processes emerge. Within the domain of empathy, pain empathy represents a prominent paradigm due to the robust and replicable elicitation of empathic responses (Singer et al. 2004). The neural mechanisms underlying pain empathy are supported by a distributed network, including the anterior cingulate cortex, supplementary motor area, anterior insula, somatosensory cortex II, inferior parietal cortex, and amygdala (Fan et al. 2011). Importantly, these regions interact dynamically through oscillatory neural activity, which coordinates the temporal and spatial processing of empathic responses.

Emerging evidence suggests that impairments in empathy in PD may stem from disruptions in dopaminergic and non‐dopaminergic pathways, as well as alterations in functional connectivity within brain networks associated with emotional and social processing (Bodden, Dodel, and Kalbe 2010). Structural and functional neuroimaging studies have highlighted reduced activity in key higher‐order social cognitive processing brain regions that are susceptible to dysfunction in neurodegenerative diseases, including the frontal lobe, anterior cingulate cortex, amygdala, and basal ganglia (Alonso‐Recio et al. 2021; Coundouris et al. 2019). However, these findings provide limited insight into the temporal dynamics and oscillatory mechanisms of empathy processing in PD. Moreover, the component of empathy impairment in PD patients remains controversial. Coundouris, Adams, and Henry (2020) suggested that patients with PD exhibit cognitively driven reductions in empathy scores with no significant alterations in affective empathy. However, recent findings were inconsistent with these assertions. A study involving event‐related potentials (ERPs) revealed a conspicuous absence of early automatic frontal (affective empathy) and late‐controlled parietal responses (cognitive empathy) related to pain in patients with PD during the off period (Hu et al. 2021).

Time–frequency analysis was used to decompose the dynamic neuronal oscillations present within continuous signals into distinct temporal and spectral components as well as evaluate the parallel processing of information in the brain (Zhang et al. 2023). Both internal psychological fluctuations and external stimuli elicit event‐related synchronization (ERS) and event‐related desynchronization (ERD). Seong‐Wook (Valentini et al. 2012)^2023^ reported that hippocampus‐dependent theta oscillations within the cingulo‐amygdala circuit in the right hemisphere of mice are an essential component of the cognitive process that drives empathic fear. At the same time, theta oscillations are also involved in processes related to emotional sharing and reward (Christie and Tata 2009; Yoshimura and Kawamura 2005). The cortical responses elicited by laser and auditory stimulation during the observation of video clips depicting either noxious or non‐noxious stimulation of a stranger's hand were analyzed using three distinct source localization methods. The results indicated that observing others' pain modulated cortical beta ERD, a response specifically associated with noxious laser stimulation, but not with auditory stimulation (Valentini et al. 2012). Yoshimasa suggested that alpha/beta oscillations underlie the sensory qualities of others' pain, whereas the gamma band reflects the cognitive aspect (Motoyama et al. 2017). Alterations in frequency‐specific neural activity in PD are indicative of varying levels of cognitive, emotional, and motor dysfunction, and these neural changes can be detected in the early stages of the disease (Conti et al. 2023; Yin et al. 2021). PD patients have abnormalities in theta and beta rhythms in the prefrontal cortex, which are closely related to cognitive and emotional components (Singh et al. 2020).

This study aims to investigate the dynamic EEG activity of empathy by comparing the changes in event‐related rhythm oscillations after the presentation of painful and neutral stimuli in patients with PD and healthy controls (HCs). To explore the EEG rhythm of pain empathy in patients with PD and further investigate the mechanism of impaired empathy in patients with PD, we focused on the frequency‐specific event‐related power associated with empathy after the presentation of painful and neutral stimuli in patients with PD and HCs, elucidating the neurophysiological mechanisms behind empathy disorders and contributing to a more comprehensive understanding of nonmotor symptoms.

## 2 Methods

### 2.1 Subjects

Twenty‐seven patients with PD were evaluated at the First Affiliated Hospital of Anhui Medical University, Hefei, China. The inclusion criteria were as follows: (1) diagnosis of idiopathic PD according to the United Kingdom Brain Bank Criteria; (2) between 40 and 80 years of age; and (3) Hoehn and Yahr disease stage ≤ 3. The exclusion criteria were as follows: (1) neurodegenerative and neuropsychological diseases other than PD; (2) difficulty understanding and cooperating with experimental procedures; (3) a change in the treatment of PD in the 2 months before the study; (4) a history of drug abuse, trauma, and/or head surgery. Clinical behavioral examinations, pain judgment tasks, and EEG collection of all PD patients were performed more than 12  h after stopping Parkinson's drugs. Thirteen HCs without neuropsychiatric disorders matched for age, sex, education, and cognitive level were also recruited through advertisements. Moreover, all subjects were not taking psychotherapy medications at the time of enrollment and signed the informed consent form. This study was supported by the Medical Ethics Committee of The First Affiliated Hospital of Anhui Medical University.

### 2.2 Experimental Task and Procedure

**FIGURE 1:**
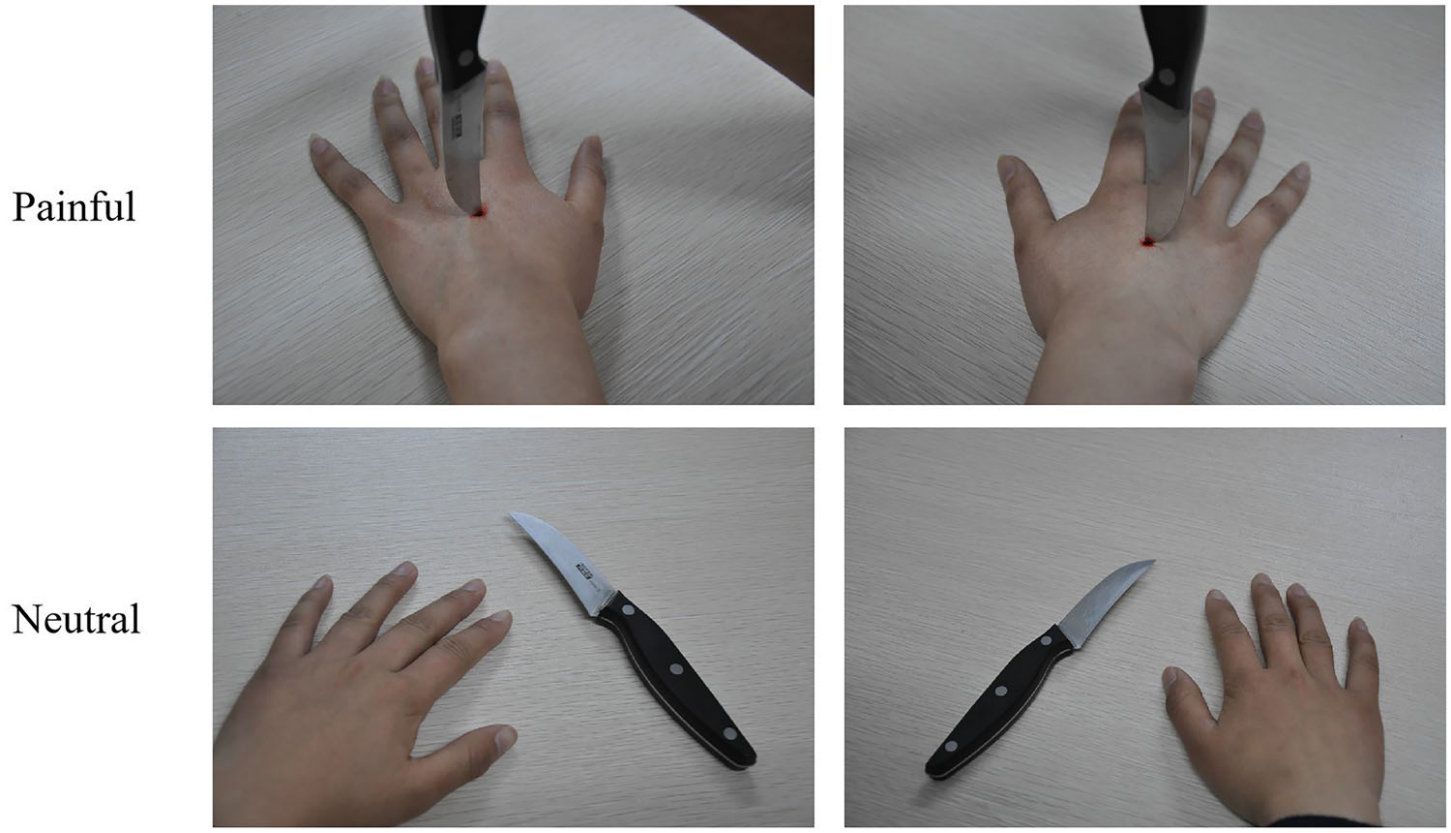
Examples of picture stimuli in the pain judgment task.

The pain empathy task was primarily a pain judgment task, in which participants were asked to determine whether the person in the picture was experiencing pain. The visual stimulation in the task involves first‐person vision, exclusively showcasing the limbs in either a painful or neutral state while omitting facial features to focus the participants' attention on the limb interactions. The stimuli consisted of photographs depicting scenarios involving left or right limbs subjected to different forms of potential harm. Specifically, these included images of limbs being pricked by needles, glass either piercing or not piercing the skin, and doors pinching or not pinching hands. For example, one of the stimulus images featured a knife either piercing or not piercing a hand (see Figure 1). This design ensured that the scenarios were relatable to everyday experiences, enhancing ecological validity.

To maintain randomness and avoid order effects, all pictures were randomly presented to each participant using E‐Prime software (Psychology Software Tools Inc., Sharpsburg, PA, USA). Subjects were presented with 20 pictures that were not presented in the study before formal recording to familiarize the subjects with the process. The experimental block consisted of 120 trials, comprising 60 neutral stimuli and 60 painful stimuli, balanced to allow for thorough assessment. Each trial commenced with presenting a fixed crisscross symbol on the screen for 500–1000 ms, serving as a preparatory cue for the upcoming stimulus. This was followed by the display of the stimulus image for 1000 ms. After the stimulus presentation, the screen would transition to a black background, prompting participants to make a judgment regarding the nature of the stimulus (painful or neutral) by pressing designated left or right buttons on a response device. This setup allowed for a clear and immediate response after viewing the images. During the task implementation, the screen refresh rate was set to 120 Hz. The visual angle of the visual stimulus was 5°. The screen brightness was set to 100 cd/m^2^ to ensure the clarity and consistency of the visual stimulus (Tian et al. 2022).

### 2.3 Electroencephalography Data Recordings and Preprocessing

Scalp EEG data were continuously recorded using 64 channels according to the international 10−20 system (Neuro Scan, Sterling, VA, USA). The entire EEG acquisition process was carried out in a room with appropriate temperature and lighting and shielded from external noise. All subjects were required to complete the EEG recording in the morning, and PD patients were also required to stop taking PD drugs for at least 12 h before EEG acquisition. During EEG recording, the sampling rate of EEG data was 500 Hz and the skin electrode impedance was maintained at < 10 kΩ. The electrode placed in the right mastoid was an online reference electrode. In addition, electrodes placed above and below the left eye were used to recognize vertical eye movements, and electrodes placed 1.5 cm outside both eyes were used to recognize horizontal eye movements.

Offline EEG data were preprocessed in combination with Matlab 2013b (Mathworks, Natick, MA, USA) and the EEGLAB 13.0b toolbox. Initially, the EEG data completed channel localization using the international 10‐05 system. Channels exhibiting a kurtosis value greater than 5 were identified as problematic and subsequently removed. Following this, spherical interpolation was applied to the remaining data. The EEG data had a bandpass filter (0.01–100 Hz), and a notch filter (48–52 Hz) was applied to remove the effects of room electricity (Gui et al. 2020). Subsequently, EEG data were epoched according to the type of stimulation; specifically, the epoch was set to 1 s before stimulation and 2 s after stimulation. Baseline correction was applied based on 1‐to‐0 s before the start of stimulation. Independent component analysis (ICA) was conducted on the epoched data to identify and remove artifact components, including those associated with blinking (localized to the forehead) and eye drifting (distributed across both sides of the forehead), as well as other artifacts, based on the characteristics of the ICA topographic maps (Jung et al. 2000). Epochs with amplitudes exceeding ±100 µV were excluded from further analysis, and the remaining EEG data were averaged to the reference.

### 2.4 Electroencephalography Data Time–Frequency Analysis

The EEG data analysis was carried out by short‐time Fourier transform with a varying Hanning time window length in the fieldtrip2017 toolbox running on a MATLAB environment. This choice was made to optimize time–frequency resolution, allowing for effective analysis of the rapidly changing brain activity associated with different stimuli. The detailed analysis method implemented multi‐taper time–frequency transformation based on multiplication in the frequency domain (Mitra and Pesaran 1999). The analysis covered a frequency range of 3–30 Hz, with 1 Hz intervals, enabling a detailed exploration of frequency‐specific information. The length of the time window was adjusted according to the analysis frequency, set to encompass three cycles of each frequency, thereby ensuring that the time resolution is sufficient to capture the dynamics of oscillatory activity while maintaining an appropriate frequency resolution (Dell'acqua et al. 2022). By using a time window length related to the period of the frequency component, a balanced time and frequency resolution can be obtained in the time–frequency analysis (Bruns 2004). The analysis time was defined from 0.2 to 1.2 s, with a temporal resolution of 2 ms. This time frame was selected to capture the immediate and subsequent neural responses to the stimuli. To correct for baseline activity, a relative change method was employed using data from 0.2 s before stimulation, ensuring that the results accurately reflect the changes induced by the stimuli rather than pre‐existing variations. By utilizing these parameters, we aimed to enhance the sensitivity and specificity of the analysis, thereby providing more reliable insights into the neurophysiological mechanisms involved in the task. Time–frequency analysis was performed on each brain channel, and the event‐related power of each channel (60 channels in total) was calculated. The values of all channels were averaged to obtain the event‐related power of the whole brain.

### 2.5 Statistical Analysis

All statistical analyses were performed using GraphPad Prism 9.5 and Matlab 2013b. According to the type of data and normality of the distribution, the demographic data and clinical characteristics of patients with PD and HCs were compared using an independent sample *t*‐test, Mann–Whitney *U* test, or chi‐square test. ANOVA was used to compare the judgment accuracy, reaction time, and event‐related delta, theta, alpha, and beta band power in the pain judgment task, with a group (PD and HCs) as between‐group factors and stimulus types (pain pictures and neutral pictures) as within‐group factors. Finally, the event‐related power of each electrode channel was correlated with the event‐related accuracy and reaction time using Pearson correlation. All multiple comparison corrections in this study were performed using the Bonferroni correction. Specifically, since event‐related power analysis was performed on four frequency bands, *p* < 0.0125 was considered statistically significant, and task‐related behavior was analyzed twice; so *p* < 0.025 was considered statistically significant. In the correlation analysis, *p* < 0.05/60 was considered statistically significant.

## 3 Results

### 3.1 Demographic and Clinical Characteristics

There were no statistically significant differences in age, gender ratio, education level, Mini‐Mental State Examination (MMSE), and Montreal Cognitive Assessment (MoCA) between the PD and HCs groups. All participants were permanent residents of East China and had the same skin color. The PD group scores were significantly higher than the HCs group with respect to Hamilton Anxiety Scale (HAMA) (*Z* = 2.305, *p *= 0.021) and Hamilton Depression Scale (HAMD) (*Z* = 2.723, *p *= 0.006). As reported in previous studies, people with the disease tend to be more prone to anxiety and depression (Table 1).

**TABLE 1:** Demographic and clinical characteristics.

	HC (*n* = 13)	PD (*n* = 27)	*p*	*t*/*χ* ^2^/*Z*
Age (years)	58.69 ± 8.61	59.18 ± 8.93	0.87	0.165^a^
Sex (female%)	6 (46.10)	9 (33.30)	0.433	0.615^b^
Education (years)	7.53 ± 3.28	6.48 ± 3.79	0.395	0.86^a^
MMSE	28.23 ± 0.92	28.18 ± 1.79	0.455	0.748^c^
MoCA	24.23 ± 2.91	23.37 ± 4.04	0.85	0.189^c^
HAMA	2.00 ± 1.95	3.74 ± 2.31	0.021	2.305^c^
HAMD	2.30 ± 1.54	5.03 ± 3.01	0.006	2.743^c^
Duration (years)		2.61 ± 2.50		
H–Y		1.5 (1, 2)		
UPDRS‐III		21.55 ± 9.21		
LEDD		294.44 ± 229.89		

*Note*: Values are expressed as the mean ± standard deviation, but the H–Y stage is described using the median and interquartile ranges.

Abbreviations: HAMA, Hamilton Anxiety Scale; HAMD, Hamilton Depression Scale; HCs, healthy controls; H–Y, Hoehn and Yahr stage; MMSE, Mini‐Mental State Examination; MoCA, Montreal Cognitive Assessment; PD, Parkinson's disease; UPDRS, unified Parkinson's disease rating scale.

^a^ Independent sample *t*‐test.

^b^ *χ* ^2^ test.

^c^ Mann–Whitney test.

### 3.2 Pain Judgment Task Results

#### 3.2.1 Task‐Related Behavioral Results

**FIGURE 2:**
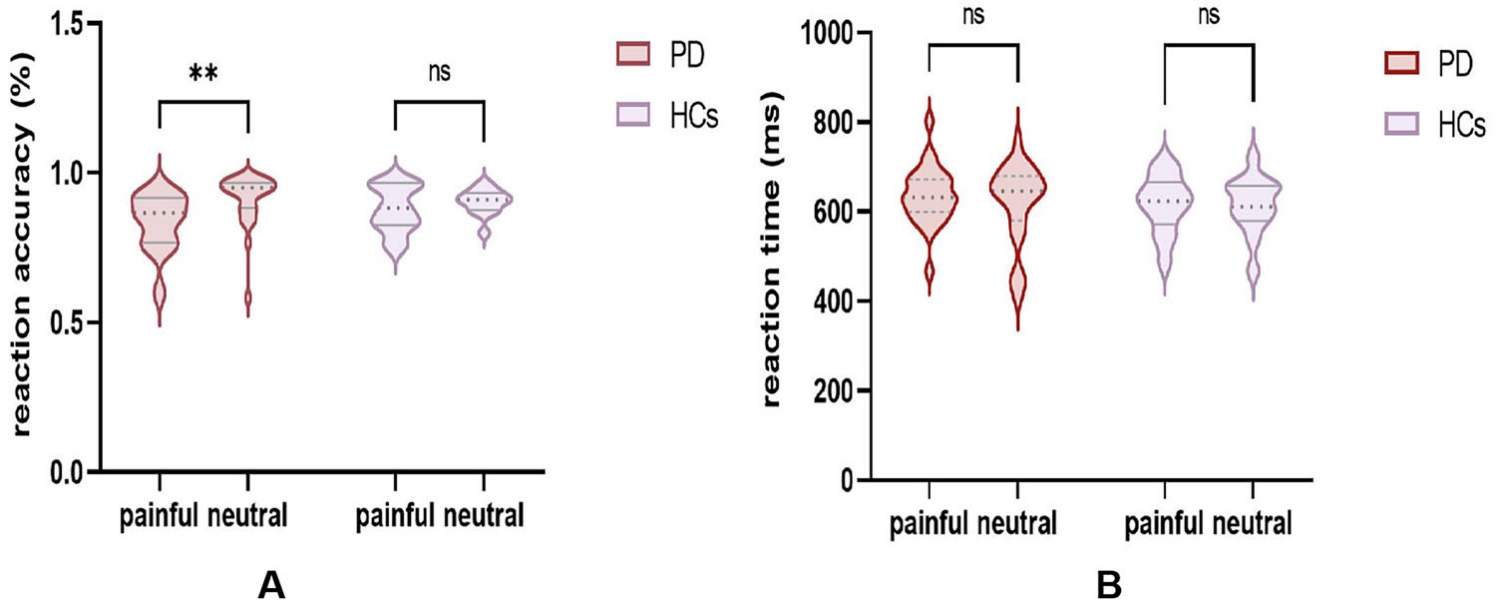
Comparison of task‐related behavioral results between patients with Parkinson's disease (PD) and healthy controls (HCs).

In the pain judgment task, there was a main effect of stimulus type on judgment accuracy (*F* = 6.73, *p *= 0.01, *p*__Bonferroni_ < 0.05, effect size = 0.08; 95% CI: [−0.006, 0.16]) but no main effect of group (*F* = 0.67, *p *= 0.41, *p*_Bonferroni_ > 0.05, effect size = 0.008; 95% CI: [−0.02, 0.03]). We observed that the judgment accuracy of painful stimuli in PD patients was significantly lower than that of neutral stimuli (*t* = 3.44, *p* = 0.0009, *p*_Bonferroni_ = 0.001, effect size = 0.93; 95% CI: [0.33, 1.52]; Figure 2A). However, there was no significant difference in judgment accuracy in HCs (*t* = 0.76, *p* = 0.44, *p*_Bonferroni_ = 0.89, effect size = 0.30; 95% CI: [−0.48, 1.07]). For event‐related reaction times, no significant main effects of group (*F* = 0.39, *p* = 0.53, *p*_Bonferroni_ > 0.05, effect size = 0.005; 95% CI: [−0.01, 0.02]) and stimulus type (*F* = 0.31, *p *= 0.57, *p*_Bonferroni_ > 0.05, effect size = 0.004; 95% CI: [−0.01, 0.02]) were observed (Figure 2B).

#### 3.2.2 Task‐Related Electroencephalography Results

**FIGURE 3:**
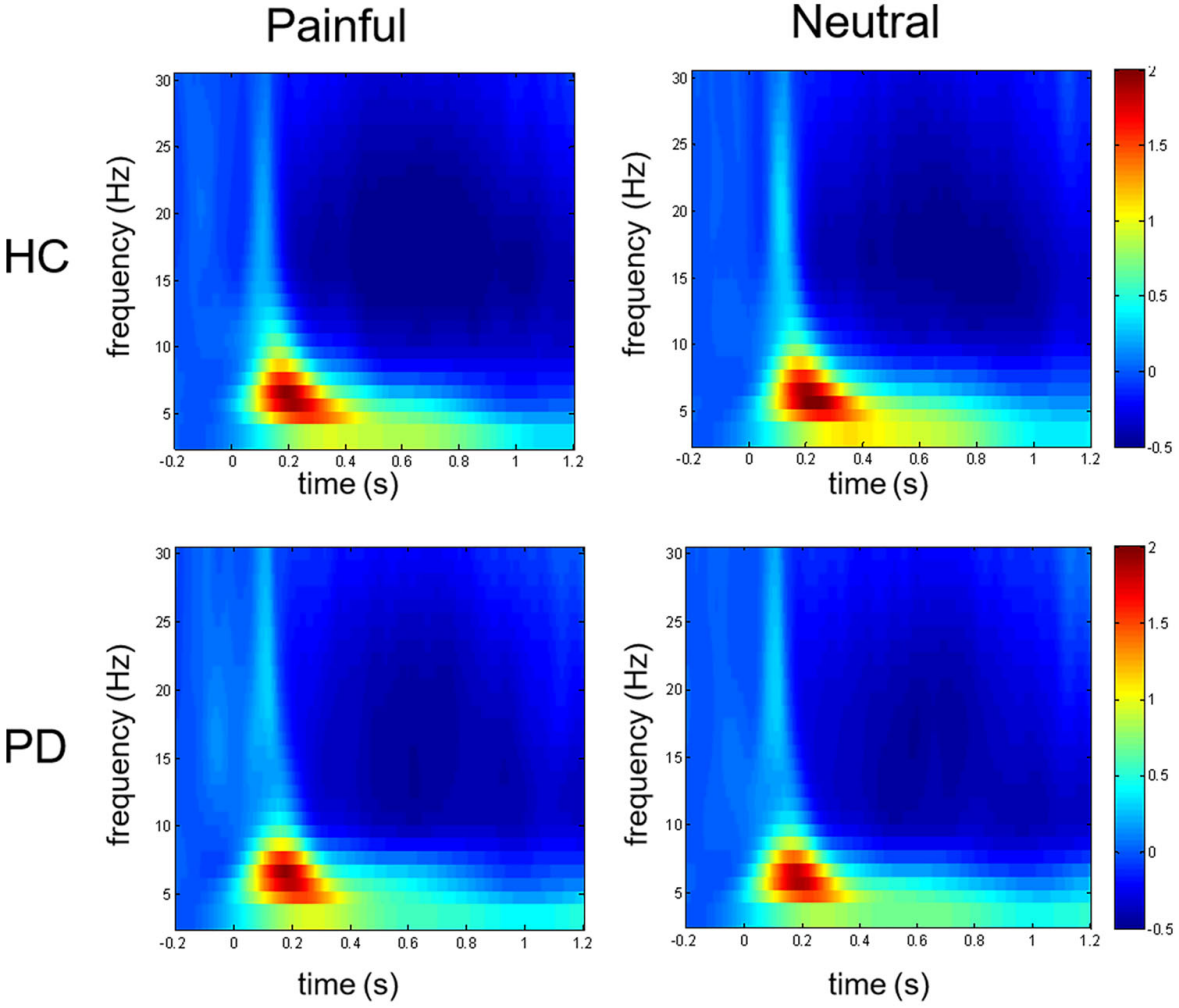
Time–frequency analysis plots of healthy controls (HCs) and Parkinson's disease (PD) patients after painful and neutral stimuli. Red represents an increase in event‐related power, and blue represents a decrease in event‐related power.

The time–frequency analysis method was used to compute the power modulation of participant responses following stimulus presentation in relation to the prestimulus power levels. Among HCs and patients with PD, the whole brain displayed that painful and neutral stimuli induced an increase in event‐related power in the theta band and a decrease in event‐related power in the beta band (Figure 3). These changes were confined to 1 s after stimulation.

**FIGURE 4:**
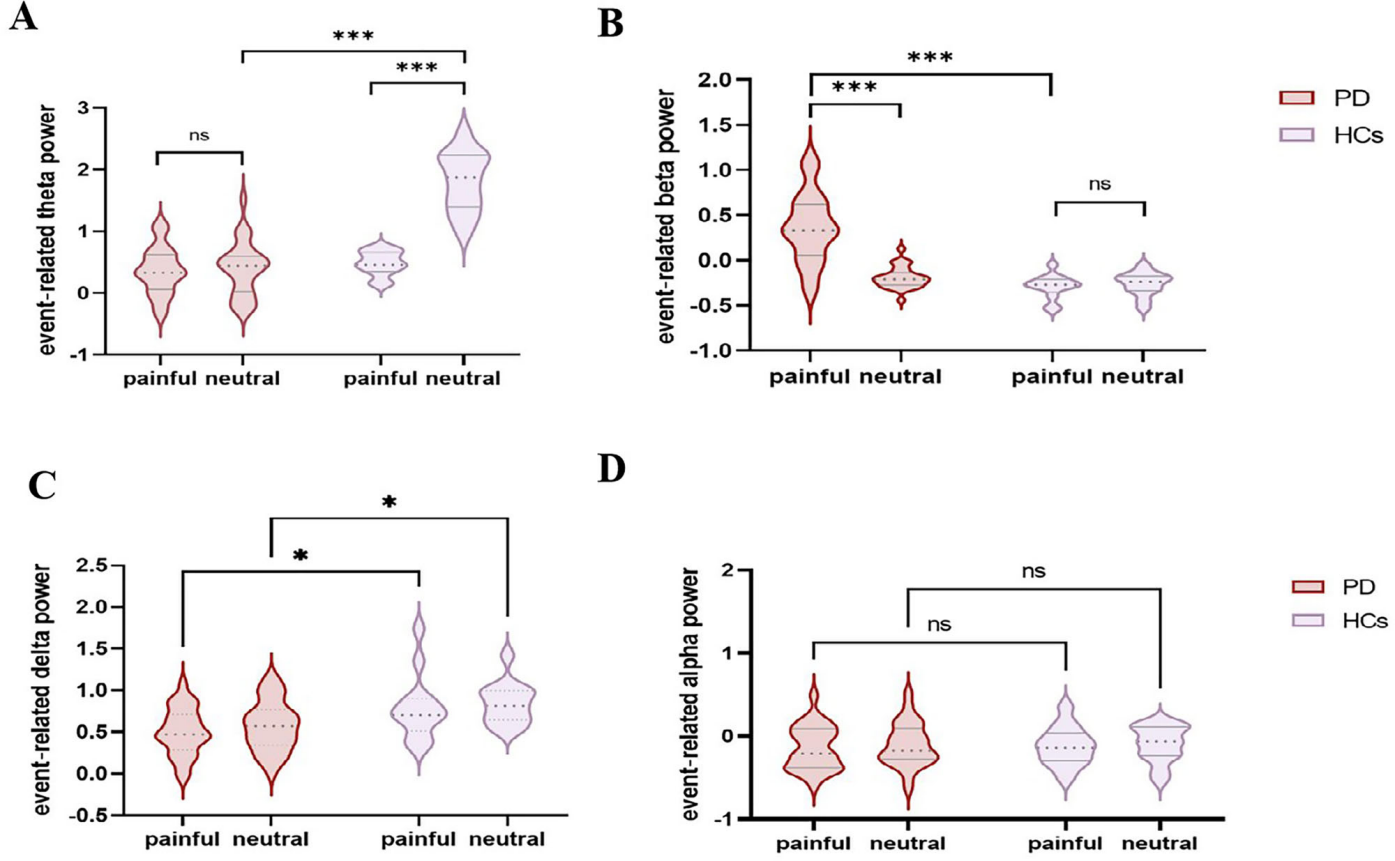
Comparison of event‐related power between patients with Parkinson's disease (PD) and healthy controls (HCs).

For event‐related theta power, we observed a significant interaction effect between group and stimulus type (*F* = 46.45, *p* < 0.001, *p*_Bonferroni_ < 0.05, effect size = 0.37; 95% CI: [0.22, 0.53]; Figure 4A). Specifically, the event‐related theta power induced by painful stimuli was significantly lower than that induced by neutral stimuli in HCs (*t* = 8.49, *p* < 0.001, *p*_Bonferroni_ < 0.001, effect size = 3.33; 95% CI: [1.79, 4.84]). However, no difference in power induced by painful and neutral stimuli was observed in PD (*t* = 0.29, *p* < 0.77, *p*_Bonferroni_ > 0.99, effect size = 0.07; 95% CI: [−0.45, 0.61]). At the same time, we also observed that the event‐related theta power in PD patients was significantly lower than that in HCs under painful stimuli (*t* = 10.53, *p* < 0.001, *p*_Bonferroni_ < 0.001, effect size = 3.55; 95% CI: [2.50, 4.58]), but no significant difference was observed under neutral stimuli (*t* = 0.89, *p *= 0.35, *p*_Bonferroni_ = 0.75, effect size = 0.30; 95% CI: [−0.36, 0.96]).

There was also a significant interaction effect of group and stimulus type in event‐related beta power (*F* = 25.02, *p* < 0.001, *p*_Bonferroni_ < 0.05, effect size = 0.25; 95% CI: [0.10, 0.38]; Figure 4B). The power induced by both painful and neutral stimuli in HCs was negative, with no significant difference (*t* = 0.32, *p* = 0.74, *p*_Bonferroni_ = 0.99, effect size = 0.12; 95% CI: [−0.64,0.89]). In PD patients, the beta power induced by painful stimuli was significantly higher than that induced by neutral stimuli (*t* = 8.30, *p* < 0.001, *p*_Bonferroni_ < 0.001, effect size = 2.26; 95% CI: [1.43, 3.06]). In addition, there was no significant difference in event‐related beta power between HCs and PD under neutral stimuli (*t* = 0.81, *p* = 0.41, *p*_Bonferroni_ = 0.83, effect size = 0.27; 95% CI: [−0.39, 0.93]), but under painful stimuli, the beta power in PD was significantly higher than that in HCs (*t* = 7.88, *p* < 0.001, *p*_Bonferroni_ < 0.001, effect size = 2.66; 95% CI: [1.76, 3.54]).

In the delta frequency band, a main effect of the group was observed (*F* = 14.57, *p* = 0.0003, *p*_Bonferroni_ < 0.05, effect size = 0.16; 95% CI: [0.04, 0.27]; Figure 4C). Specifically, alpha power induced by both painful (*t* = 2.76, *p *= 0.007, *p*_Bonferroni_ = 0.01, effect size = 0.93; 95% CI: [0.23, 1.62]) and neutral stimuli (*t* = 2.63, *p* = 0.010, *p*_Bonferroni_ = 0.02, effect size = 0.88; 95% CI: [0.19, 1.57]) was significantly lower in PD patients than in HCs. No statistically significant main effects of group(*F* = 0.07, *p *= 0.77, *p*_Bonferroni_ > 0.05, effect size = 0.001; 95% CI: [−0.008, 0.01]) or stimulation type group (*F* = 0.28, *p *= 0.59, *p*_Bonferroni_ > 0.05, effect size = 0.004; 95% CI: [−0.01, 0.02]) were observed in the alpha band (Figure 4D).

### 3.3 Correlation Analysis

**FIGURE 5:**
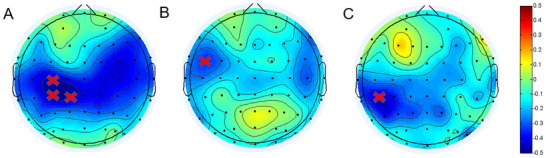
_i_ .Correlation between the event‐related power of the beta band and task‐related behavioral measures for all subjects. The beta band power was negatively correlated with the accuracy (A) and reaction time under painful stimuli (B); the power of the beta band was negatively correlated with the accuracy of neutral stimuli(C). “×” denotes pBonferroni 〈 0.05.

In the correlation analysis, the Pearson correlation between the event‐related power of each channel and the task‐related behavior was calculated. In all subjects, the beta power induced by painful stimuli was significantly negatively correlated with the accuracy (channel = C3, CP3, CP1; *r* = −0.46, *p*_Bonferroni_ < 0.05; Figure 5A) and reaction time (channel = FC5; *r* = −0.4358, *p*_Bonferroni_ < 0.05; Figure 5B). The beta power induced by neutral stimuli was also significantly negatively correlated with the accuracy of neutral stimuli (channel = CP5; *r* = −0.4493, *p*_Bonferroni_ < 0.05; Figure 5C).

## 4 Discussion

In the current study, we used the time–frequency analysis method in ERP technology to explore the neuro‐electrophysiologic changes in pain empathy among PD patients. After the presentation of painful and neutral stimuli, patients with PD showed the theta ERS and beta ERD. Patients with PD exhibited smaller beta ERD in response to painful stimuli and induced weaker theta ERS in response to neutral stimuli compared to HCs. Abnormal beta ERD differences between painful and neutral stimuli were found in PD patients, whereas theta ERS differences were absent. There was a clear correlation between changes in pain‐related beta oscillations and the accuracy of pain judgment in PD. The current study investigated the dynamic brain characteristics associated with empathy impairment in patients with PD and provided a theoretical basis for improving empathy ability.

Dopamine denervation in patients with PD affected brain networks related to social cognitive functions (Zaki 2020). Early‐ and middle‐stage PD patients already have impaired pain empathy, including both emotional empathy and cognitive empathy. Affective empathy is mainly mediated by orbital frontostriatal circuitry, while the cognitive component is related to dorsolateral frontostriatal circuitry. Dopamine depletion within the most dorsolateral part of the caudate nucleus head and striatum in patients with PD directly affects the empathy circuit, leading to its dysfunction (Gallagher, Raffone, and Aglioti 2024; Bodden, Dodel, and Kalbe 2010). Changes in theta and beta oscillations in the caudate nucleus and dorsolateral prefrontal cortex may contribute to cognitive impairment in PD (Singh et al. 2020; Paulo et al. 2023). The decrease in dopamine in patients with PD increases beta oscillations in cortico‐striato‐thalamo‐cortical circuits, which decreases beta ERD during movement preparation and during exercise, ultimately leading to symptoms of rigidity and bradykinesia (Iskhakova et al. 2021). Theta oscillations originate from the anterior cingulate cortex and hippocampus (Buzsáki 2002; Pizzagalli, Oakes, and Davidson 2003) and are very sensitive to the dynamic processing of emotional stimuli. According to Dell'acqua et al. 2022), beta ERD occurs when watching one's own objects being stimulated with pain. The asynchronous occurrence of beta oscillations is considered a feature of cortical sensorimotor processing, particularly when observing noxious physical events (Riečanský and Lamm 2019). In the present study, beta ERD induced by painful pictures and theta ERS induced by neutral stimulation were significantly weaker in PD patients than in HCs. In addition, the central parietal pain‐related beta wave power in PD patients was significantly negatively correlated with the accuracy of painful stimulation, suggesting that the reduction of beta ERD corresponds to the reduction of pain judgment accuracy. Reduced beta ERD responses to pain may mediate the process of pain empathy impairment in patients with PD, which aligns with previous research highlighting the crucial role of beta oscillations in pain processing (Hauck, Lorenz, and Engel 2008). Theta oscillations are sensitive in exploring motivation‐related reward mechanisms, as evidenced by reward‐related theta oscillatory activity in the right frontal cortex during gambling games (Christie and Tata 2009). Event‐related theta ERD has also been shown to be a potential neurologic marker of apathy severity (Zhu et al. 2019). In the current study, theta ERS induced by neutral stimuli was significantly reduced in patients with PD compared to HCs, which may reflect the lack of motivation in patients with PD. This decrease in motivation may also affect pain empathy processes.

In addition to neurotransmitter dysregulation, motor‐emotion coupling disorders may also contribute to impaired empathy in PD patients. Motor imitation can promote social interaction and is widely considered to be an indicator of mirror neuron activation (Cracco et al. 2018). Cognitive and motor impairment mediated by abnormal beta oscillations can cause empathy impairment in PD patients through mechanisms such as automatic imitation (Cracco et al. 2018). Drawing from prior studies on facial emotion recognition, simulation theory posits that emotion recognition proceeds by simulating internally generated somatosensory representations triggered by perceived actions (Argaud et al. 2018). Overall, impairments in motor processing engender deficits in emotion recognition (Marneweck and Hammond 2014; Wood et al. 2016; Verreyt et al. 2011). Simulation theory also occurs during pain empathy. Beta rhythm may be a neurophysiological indicator of human mirror neuron system activity (Muthukumaraswamy and Johnson 2004), reflecting input from mirror neurons and is closely related to thalamocortical excitability (Steriade and Llinás 1988). Watching others in pain induces sensorimotor resonance, and this empathic resonance depends on the perceived similarity between the observer and the observed (Avenanti, Sirigu, and Aglioti 2010). Reduction in beta ERD reflects suppression of motor preparation in pain empathy (Valentini et al. 2012). Consequently, the beta rhythm, conventionally associated with motor symptoms (Guerra et al. 2022), also assumes significance as a nonmotor symptom rhythm (Paulo et al. 2023).

The ability of individuals with PD to distinguish between painful and neutral stimuli in the context of pain empathy is significantly diminished. In this study, HCs exhibited significantly lower theta power in response to pain compared to neutral stimuli; however, this difference was absent in the PD group. Similarly, while a difference in beta power between pain‐related and neutral‐related stimuli was observed in PD, no such distinction was found in HCs. In the process of emotional processing, temporal variations in theta and beta oscillations are associated with specific implications. It is generally believed that an increase in theta oscillation within 300 ms after stimulus presentation is related to automatic orientation and an increase after 300 ms is related to top‐down processing of salient stimuli. The decrease in theta oscillations from 1000 to 4000 ms is related to reappraisal, which is a reduction in stimulus priority through selective attention (Uusberg, Thiruchselvam, and Gross 2014; Thiruchselvam et al. 2011). The latency of beta ERD is usually associated with involuntary attentional reorientation, context updating, and conscious evaluation, and in particular, 350–450 ms after the stimulus was indeed compatible with the interaction of top‐down and bottom‐up attentional processes (Polich 2007; Valentini et al. 2011). The above indicates that PD patients have weak orientation and difficulty distinguishing between neutral and painful images, which makes them more likely to judge painful images as neutral images. Carola (Jung et al. 2000)^2022^ also concluded that dysphoric patients lack later conscious and adaptive regulation of unpleasant stimuli compared with controls.

Theory of mind is essential in inferring the mental states of others mediated by a complex neuroanatomic network involving the medial prefrontal cortex (Frith and Frith 1999). Corticostriatal connections may be compromised to varying degrees by dopamine deficiency or amygdala pathology, which mediates theory of mind deficits in neurodegenerative diseases, such as PD and Huntington's disease (Argaud et al. 2018; Bodden, Dodel, and Kalbe 2010; Brüne et al. 2011). Somatosensory recruitment may act as a compensatory mechanism after dopamine depletion or amygdala pathology, recalling emotion‐related knowledge through the somatosensory cortex and overcoming the emotional deficits in patients with PD (Argaud et al. 2018; Adolphs, Tranel, and Damasio 2003). An analysis of EEG source localization in fear expression recognition showed that event‐related responses in patients with PD originate from the parietal somatosensory cortex while originating from the amygdala and temporal lobe in HCs (Yoshimura et al. 2005). The occurrence of PD is closely related to the damage of the basal ganglia‐thalamus‐cortex circuit. In PD, after the loss of midbrain dopaminergic neurons, dopamine dysfunction will lead to an imbalance in the regulation between the direct pathway and the indirect pathway (Perovnik et al. 2023). Its compensatory mechanism may lead to the rearrangement of the brain activation pattern (Argaud et al. 2018). Observing pain in others suppresses motor cortical excitability and modulates neural activity elicited by concomitantly delivered nociceptive somatosensory stimulation (Morrison et al. 2013). The somatosensory cortex is activated in response to observing the actions of others and anticipating the painful consequences of interacting with noxious objects and is more activated in response to mirror‐touch or mirror‐pain synesthesia. The somatosensory cortex also has an important function in empathy (Riečanský and Lamm 2019). PD patients have abnormally increased beta‐gamma phase‐amplitude coupling in the sensorimotor cortex due to neural synchronization in the basal ganglia, resulting in uncoordinated brain activity (Hodnik et al. 2024).

This study also has some limitations. First, previous studies have proven that dopamine deficiency can impair social cognitive circuits, but the impact of dopamine on pain empathy in PD was not explored in this study. Second, this study used static pictures as stimuli. While dynamic stimuli might better reflect daily life, dynamic videos could overwhelm patients with PD by increasing cognitive load and distraction. Future research should integrate dynamic stimuli to enhance ecological validity while maintaining comparability. Finally, the sample size gap between the patients with PD and HCs was relatively large. The lack of detailed demographic information (such as socioeconomic status, smoking and drinking status) is also a limitation of this study. Although there is no difference between the two groups of participants with respect to demographic information, future research will continue to increase the sample size and improve demographic information to be more comprehensive and explore the brain activation mechanism underlying pain empathy in patients with PD.

In conclusion, PD disrupts the typical neural oscillatory mechanisms underlying pain empathy, notably impairing the ability to discriminate between painful and neutral stimuli and altering the synchronization–desynchronization dynamics essential for accurate pain processing. Dysregulation of theta and beta oscillations appears to mediate the observed deficits in pain empathy among individuals with PD. A deeper understanding of these oscillatory alterations could advance our knowledge of the neurophysiological basis of empathy impairments in PD and identify potential biomarkers and therapeutic targets to enhance empathic functioning in this population.

## Author Contributions

**Jinying Han**: conceptualization, methodology, software, data curation, investigation, validation, visualization, writing–original draft, writing–review and editing, project administration, formal analysis. **Liuzhenxiong Yu**: conceptualization, investigation, writing–original draft, writing–review and editing, methodology, validation, software, data curation. **Xin Chen**: conceptualization, investigation, methodology, validation, writing–review and editing, software, data curation. **Mengqi Wang**: conceptualization, investigation, methodology, validation, data curation, software. **Ziye Zhao**: conceptualization, investigation, methodology, software, data curation. **Pingping Liu**: conceptualization, methodology, software, investigation, data curation. **Lili Hu**: conceptualization, methodology, software, data curation. **Lingling Lv**: conceptualization, methodology, investigation, validation. **Fengbo Xing**: conceptualization, methodology, software. **Ruihua Cao**: conceptualization, methodology, investigation. **Rong Ye**: conceptualization, methodology, software. **Kai Wang**: funding acquisition, writing–review and editing, project administration, supervision, resources, validation. **Panpan Hu**: writing–review and editing, visualization, project administration, resources, supervision, funding acquisition, validation, methodology, conceptualization.

## Conflicts of Interest

The authors declare no conflicts of interest.

### Peer Review

The peer review history for this article is available at https://publons.com/publon/10.1002/brb3.70294.

## Data Availability

Raw data can be obtained by contacting the corresponding author.
